# Evaluation of a Point-of-Care Ultrasonography Decision-Support Algorithm for the Diagnosis of Transient Synovitis in the Pediatric Emergency Department

**DOI:** 10.1001/jamanetworkopen.2021.16915

**Published:** 2021-07-13

**Authors:** Marah Zoabi, Noa Kvatinsky, Itai Shavit

**Affiliations:** 1 Rappaport Faculty of Medicine, Technion-Israel Institute of Technology, Haifa, Israel; 2 Pediatric Emergency Department, Rambam Health Care Campus, Haifa, Israel

## Abstract

This case series evaluates the performance of a point-of-care ultrasonography (POCUS) decision-support algorithm for the diagnosis of transient synovitis in the pediatric emergency department (ED).

## Introduction

Transient synovitis (TS) is a self-limiting disease characterized clinically by acute hip pain. The diagnosis is confirmed by excluding other severe diseases, such as septic arthritis, osteomyelitis, and Legg-Calvé-Perthes disease. Ultrasonography examination frequently reveals hip effusion.^1,2^

A point-of-care ultrasonography (POCUS) decision-support algorithm (DSA) is regularly used to identify TS in the pediatric emergency department (ED) of Rambam Health Care Campus, a tertiary hospital in Haifa, Israel.^2,3^ The POCUS-DSA includes a set of 5 clinical criteria that must be met, followed by bedside ultrasonography examination of the hip joint (Figure).^1,2^ Here, we evaluate the performance of the POCUS-DSA in the diagnosis of TS among children presenting to the pediatric ED.

**Figure.**
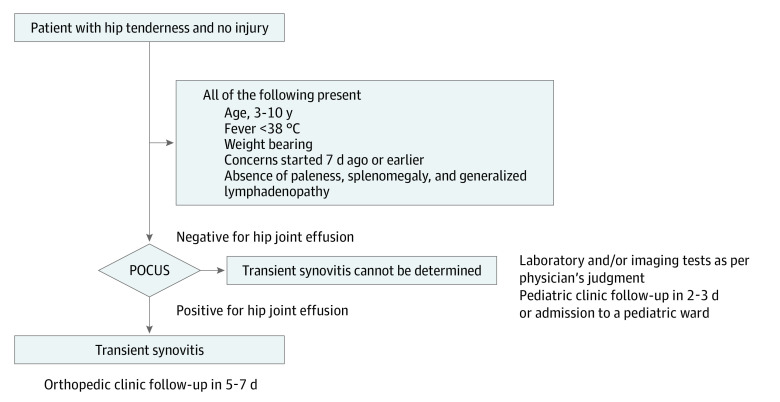
Point-of-Care Ultrasonography (POCUS) Decision-Support Algorithm to Identify Transient Synovitis in Children Aged 3 to 10 Years Who Presented With Nontraumatic Hip Tenderness to the Emergency Department

## Methods

This study was approved by the Rambam Health Care Campus ethics committee. Informed consent was not necessary because of the retrospective nature of the study, in accordance with Rambam Health Care Campus policy. The reporting guideline for case series was used for this study.

This case series analyzed all children with nontraumatic hip tenderness who met the POCUS-DSA criteria between January 1, 2014, and December 31, 2019. Ultrasonography was performed using an accepted technique.^2^ Patients with hip effusion received a diagnosis of TS in the ED and were scheduled for a follow-up visit in 5 to 7 days. Patients without hip effusion were assessed with laboratory or imaging tests, as per clinician judgment (Figure).^2^ A final diagnosis of TS was made for any patient who had spontaneous resolution of symptoms and a reduction in the amount of effusion at the follow-up visit.^1^ Sensitivity, specificity, positive predictive value (PPV), negative predictive value (NPV), positive likelihood ratio (LR), and negative LR were used to assess the accuracy of the POCUS-DSA. Data were analyzed using StatsDirect statistical software version 2.6.6 (StatsDirect Ltd). Data analysis was performed from October to November 2020.

## Results

Overall, 1461 children with nontraumatic hip tenderness attended the ED during the study period. POCUS-DSA was applied to 621 patients (429 boys [69%]; 192 girls [31%]) with a mean (SD) age of 5.5 (1.9) years. Patients were examined by 38 ED physicians who verified the inclusion POCUS-DSA clinical criteria and performed all the examinations. The study cohort included 6 patients with septic arthritis, 4 with osteomyelitis, 4 with Legg-Calvé-Perthes disease, and none with malignant entities or other serious conditions. In the ED, 539 patients were correctly diagnosed as having TS, 22 were correctly diagnosed as not having TS, and 54 were misdiagnosed as not having TS but correctly diagnosed at follow-up visits. Six ED patients were misdiagnosed as having TS but were found to have another condition at follow-up visits; all of them recovered completely (Table). The accuracy of POCUS-DSA for the diagnosis of TS was as follows: sensitivity, 90.9% (95% CI, 88.3%-93.1%); specificity, 78.6% (95% CI, 60.5%-89.8%); PPV, 98.9% (95% CI, 97.6%-99.5%); NPV, 28.9% (95% CI, 20.0%-40.0%); positive LR, 4.25 (95% CI, 2.1-8.6); and negative LR, 0.12 (95% CI, 0.08-0.16). Forty-eight patients (7.8%) without hip effusion underwent blood tests.

**Table.** Demographics, Duration of Limp, Ultrasonography Findings, Diagnosis, and Disease Course in Patients Who Were Missed by the POCUS Decision-Support Algorithm

Patient No.	Age, y	Sex	Duration of limp before ED arrival, d	POCUS findings of the hip joint	Diagnosis	Disease course
1	10	Male	1	Bilateral effusion, right greater than left	Patellar tendinitis	Diagnosed at the outpatient clinic first visit. Treated with NSAIDS with complete recovery.
2	10	Male	6	Left side effusion	Arthralgia of the knee joint	Diagnosed at the outpatient clinic first visit. Treated with NSAIDS with complete recovery.
3	7	Male	5	Right side effusion	Legg-Calvé-Perthes disease	Diagnosed at the outpatient clinic third visit. Treated operatively with epiphysiodesis of the greater trochanter by drilling and curettage. At the 1-year follow-up visit, the parents reported a complete recovery.
4	6	Male	1	Left side effusion	Septic arthritis	Brucellosis was diagnosed. Treated with antibiotics with complete recovery.
5	5	Female	7	Left side effusion	Osteoid osteoma of the femur	Diagnosed at the orthopedic outpatient clinic first visit. Treated with computed tomography–guided radiofrequency ablation. At the 1-year follow-up visit, the parents reported a complete recovery.
6	3	Male	2	Right side effusion	Legg-Calvé-Perthes disease	Diagnosed at the orthopedic outpatient clinic first visit. Treated conservatively. At the 1-year follow-up visit, the parents reported a complete recovery.

Abbreviations: ED, emergency department; NSAIDS, nonsteroidal anti-inflammatory drugs; POCUS, point of care ultrasonography.

## Discussion

The findings of this study suggest that the POCUS-DSA accurately identified TS, as reflected by the high sensitivity and PPV. The positive LR of 4.25 suggests that the POCUS-DSA can be helpful in ruling in the diagnosis of TS. Hip effusion may not always be present in TS at presentation.^4^ This is reflected by the limited specificity and low NPV, which suggest that the POCUS-DSA cannot reliably rule out TS. All six patients who were misdiagnosed as having TS were correctly diagnosed at follow-up visits, and all completely recovered. These data provide evidence for the safety of the POCUS-DSA.

To our knowledge, this study is the first to suggest a rule-in diagnostic tool for TS, instead of one of exclusion. The traditional approach advocates the use of laboratory tests to differentiate between TS and other serious diseases.^4,5^ In our cohort, only 7.8% of the children underwent blood tests. Avoiding unnecessary blood tests by using the POCUS-DSA can reduce the number of children who undergo venipuncture and minimize pain and distress.^6^ Possible limitations of this study include its retrospective nature, the operator-dependent nature of the ultrasonography examination, the absence of an objective reference standard, and the lack of external validation of the POCUS-DSA.
